# Study of ZnS Nanostructures Based Electrochemical and Photoelectrochemical Biosensors for Uric Acid Detection †

**DOI:** 10.3390/s17061235

**Published:** 2017-05-28

**Authors:** Yao Zhao, Xueyong Wei, Niancai Peng, Jiuhong Wang, Zhuangde Jiang

**Affiliations:** State Key Laboratory for Manufacturing Systems Engineering, Xi’an Jiaotong University, Xi’an 710054, China; zhaoyao@stu.xjtu.edu.cn (Y.Z.); pnc@mail.xjtu.edu.cn (N.P.); jhw@xjtu.edu.cn (J.W.); zdjiang@mail.xjtu.edu.cn (Z.J.)

**Keywords:** biosensor, enzyme, nanostructures, ZnS, UA

## Abstract

Uric acid (UA) is a kind of purine metabolism product and important in clinical diagnosis. In this work, we present a study of ZnS nanostructures-based electrochemical and photoelectrochemical biosensors for UA detection. Through a simple hydrothermal method and varying the ratio of reaction solvents, we obtained ZnS nanomaterials of one-dimensional to three-dimensional morphologies and they were characterized using field emission scanning electron microscopy (FESEM) and X-ray diffraction (XRD). To fabricate the UA biosensor and study the effect of material morphology on its performance, ZnS nanomaterials were deposited on indium tin oxide (ITO) conducting glass and then coated with uricase by physical absorption. Three kinds of working electrodes were characterized by cyclic voltammetry method. The effect of material morphology on performance of UA detection was investigated via amperometric response based electrochemical method based on enzymatic reaction. The ZnS urchin-like nanostructures electrode shows better sensitivity compared with those made of nanoparticles and nanoflakes because of its high surface-area-to-volume ratio. The photoelectrochemical method for detection of UA was also studied. The sensitivity was increased 5 times after irradiation of 300 nm UV light. These results indicate that ZnS nanostructures are good candidate materials for developing enzyme-based UA biosensors.

## 1. Introduction

Uric acid (UA) is a kind of purine metabolism product and important in clinical diagnosis. For a healthy adult male, the normal range of UA in serum is 149∼416 μM, higher than that of adult females 89∼357 μM and children 180∼300 μM [1]. UA concentration exceeding the health standard will lead to gout [2] or other cardiovascular disease [3].Therefore, it is very important to detect the concentration of UA in human body or human waste for the early diagnosis of certain kinds of disease related to purine metabolism in clinic [4]. In recent years, various UA biosensors using nanomaterials and nanostructures have been reported due to their high effective surface area, improvement of mass transport and good conductivity [5,6,7,8,9,10,11,12]. Metal materials like gold nanoparticles [13] exhibit excellent electron transfer efficiency between enzyme and electrode surface, which leads to wide applications in electrochemical biosensors. Semiconductor nanomaterials also received considerable attentions in the fields of both electrochemical and photoelectrochemical biosensors. For example, Zinc based nanomaterials (mainly including ZnO and ZnS) are a series of wide bandgap (3.37 eV, 3.68 eV) II–VI semiconductor materials with special properties. Several methods to prepare zinc nanomaterials such as thermal evaporation and chemical vapor deposition process [14] have been investigated considering their wide applications in optics [15], electronics [16], biosensor [17] and etc. Hydrothermal method is widely used to prepare zinc nanomaterial due to its simplicity and high efficiency. By this method zinc based nanostructures including ZnS:Cu/ZnS nanoparticles [18], ZnO nanowires [19] and nanoflakes [5] have been synthesized.

In this paper, we present a study of ZnS nanostructures-based electrochemical and photoelectrochemical biosensors for UA detection. A hydrothermal method was employed to synthesize ZnS nanomaterials with controllable morphology of one to three dimensions and a simple spin-coating method to fabricate UA biosensor without using any organic link molecule [20]. The terpilenol was used to combine the ZnS nanomaterials on ITO glass and was then evaporated. Uricase was immobilized to ZnS nanomaterials via entrapment method. In photoelectrochemical method, light was used to excite the active species on the electrode while current was obtained as detection signal. Photoelectrochemical method usually has higher sensitivity and low background signals compared with traditional electrochemical method. Several ZnS nanomaterial based photoelectrochemical biosensors have been reported [21,22,23]. However, this is the first time a ZnS based biosensor for UA detection has been presented using the photoelectrochemical method.

## 2. Experimental Details

### 2.1. Reagents

Anhydrous zinc acetate (Zn(CH${}_{3}$COOH)${}_{2}$, 99.99%), ethylenediamine (C${}_{2}$H${}_{8}$N${}_{2}$, 99%), thiourea (CH${}_{4}$N${}_{2}$S, 99.5%), ascorbic acid (AA), UA, urea and glucose were purchased from Aladdin, China. Uricase (EC 1.7.3.3) and Nafion (5 wt %) were purchased from Sigma-Aldrich (Shanghai, China). All reagents were used without further purification.

### 2.2. Synthesis of ZnS Nanostructures and Characterization

ZnS nanocrystals were synthesized by a modified process based on the previous work in the literature [24]. First, 0.5 mmol of Zn(CH${}_{3}$COOH)${}_{2}$ and 1 mmol of CS(NH${}_{2}$)${}_{2}$ were dissolved in 40 mL solvent consist of *x*C${}_{2}$H${}_{8}$N${}_{2}$ to (100-x) H${}_{2}$O. The solution was transferred into a 50 mL Teflon-lined autoclave, and then heated in oven at 160 ${}^{\circ }$C for 10 h. After cooling to room temperature, the synthesized nanomaterials were purified by centrifugation and washing with ethanol repeatedly and then dried under vacuum over night. The morphology and nanocrystal type of the nanomaterials were characterized by a field emission scanning electron microscopy (FESEM, Su-8010, Hitachi, Tokyo, Japan) and X-ray diffractometer (XRD, D8 advance, Bruker, Karlsruhe, Germany) in the range of 20${}^{\circ }$∼80${}^{\circ }$ with a scanning speed of $8^{\circ }/\min$. Absorption and photoluminescence spectra were tested by UV-visible spectrophotometer (UV 3600, Shimadzu, Kyoto, Japan) and fluorescence spectrofluorometer (FluoroMax, Horiba, Paris, France) using an excitation wavelength of 310 nm.

### 2.3. Fabrication of Biosensors and Measurements

To fabricate the biosensor electrode, 0.05 g of nanomaterial was mixed uniformly with 0.3 mL terpineol. The mixture was then spin-coated on ITO conducting glass (1 cm × 0.5 cm) at a speed of 300 rpm. The electrode was heated at 100 ${}^{\circ }$C in vacuum for 12 h. To immobilize the enzyme onto the electrode, the nanomaterials coated electrode was firstly wetted by PBS solution. After being dried by N${}_{2}$ gas, 20 μL of 1 mg/mL uricase was dropped onto the electrode. The loosely bonded enzyme was removed by immersing the electrode in PBS solution for 30 min. Finally, 10 μL of Nafion solution was added to the electrode to protect the uricase from leakage. The electrochemical experiments were done by a conventional three electrode analyzer (CHI660E) while the nanomaterials and enzyme decorated ITO substrate as working electrode. The photoelectrochemical tests were conducted in a dark box with a 300 nm UV lamp (WFH-204B) inside.

## 3. Results and Discussion

### 3.1. Characterization of ZnS Nanostructures

**Figure 1:**
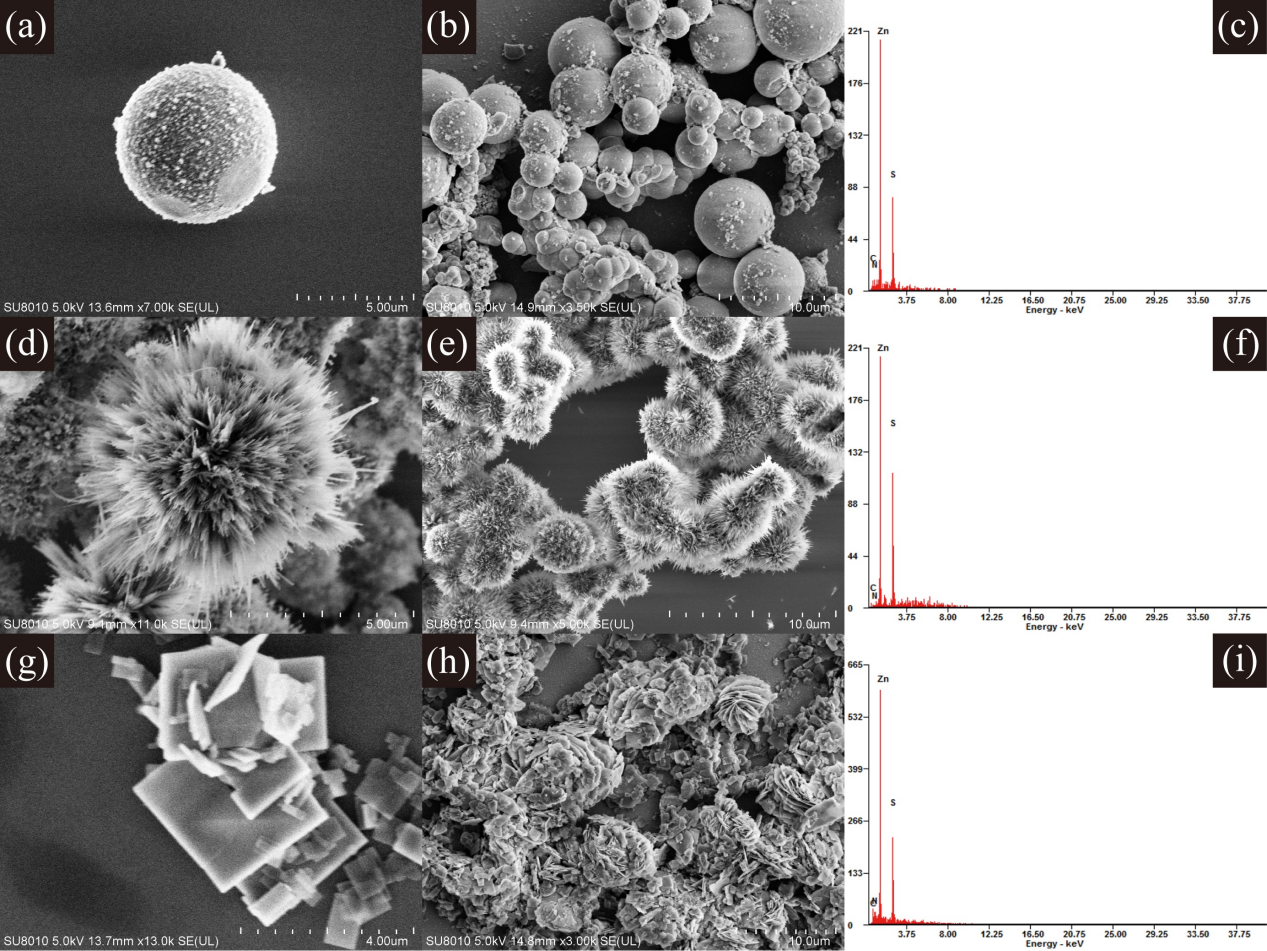
Scanning electron microscopy (SEM) and energy dispersive X-ray spectroscopy (EDX) results of ZnS nanostructures synthesized with different C${}_{2}$H${}_{8}$N${}_{2}$:H${}_{2}$O ratios of (**a**–**c**) 0:1; (**d**,**f**) 1:1 and (**g**–**i**) 3:1.

The morphologies of products fabricated with different molar ratios of ethylenediamine and water in the hydrothermal process were characterized by SEM. The SEM images in Figure 1 show that the solvent ratio of ethylenediamine and water has a crucial effect on the morphology of the ZnS nanostructures [25,26]. When the ratio is 0:1, the resultant are ZnS nanoparticles with a size range of 2∼5 μm. When the ratio is changed to 1:1, the observed ZnS nanocrystal is urchin-like spherical structures assembled into bunches of nanorods of approximately 2∼3 μm in length. The synthesized product turns to be nanoflakes with a side length of around 3 μM at the ratio of 3:1. EDX results in Figure 1c,f,i indicate that all the synthesized nanostructures consist of Zn and S elements. Ethylenediamine plays the role of a molecular template in effecting the final crystallization of ZnS nanostructures [25,26]. Therefore, the critical point to get controllable ZnS nanostructures is to vary the ratio of ethylenediamine to water in the hydrothermal process.

**Figure 2:**
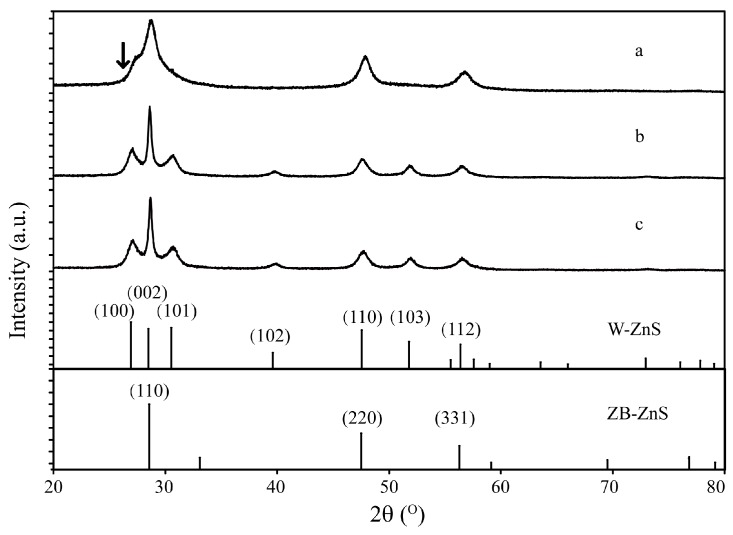
X-ray diffracion (XRD) patterns of ZnS nanostructures synthesized with different C${}_{2}$H${}_{8}$N${}_{2}$:H${}_{2}$O ratios of (**a**) 0:1; (**b**) 1:1 and (**c**) 3:1.

The crystallinity of the ZnS nanostructures was characterized by XRD. The diffraction peaks in Figure 2a indicate that the synthesized ZnS nanoparticles are mainly of zinc-blende structure in accord with the reported data (JCPDS File No. 05-0566). There is also a trace of wurtzite structure in the ZnS nanoparticles with a very weak peak at $27.32^{\circ }$ shown by the arrow, indicating the diffraction of the (100) plane of the wurtzite structure. The diffraction peaks in Figure 2b,c show that the ZnS urchin-like nanostructures and nanoflakes have a wurtzite structure according to the standard card (JCPDS File No. 75-1534).

**Figure 3:**
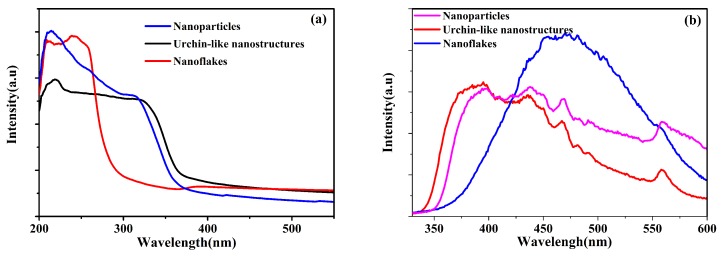
(**a**) UV-visible and (**b**) photoluminescence spectra of the synthesized nanocrystals.

Absorption spectra of synthesized ZnS nanocrystals are shown in Figure 3a. It can be seen that the products with different morphologies have absorption edges at 244 nm, 314 nm and 320 nm respectively which were calculated by finding the crossing points of *x*-axis and the tangent line of the absorption cures with largest slope. The high absorption values in the UV area also confirms the existence and purity of ZnS nanocrystals. Figure 3b shows the photoluminescence spectra excited at a wavelength of 310 nm. The fluorescence peak at 380 nm was due to the hole traps originated from unsaturated $sp^{3}$ orbital of the surface S atoms, while the defect emission at 470 nm mainly originates from point defects induced by the isolated Zn vacancies [27,28,29].

### 3.2. Electrochemical Characterizations and Measurements

Regarding UA detection, uricase was immobilized on the biosensor electrode made of ZnS nanostructures by electrostatic adsorption. The enzymatic reaction of UA that took place on the Nafion/Uricase/ZnS/ITO electrode are presented as follows [5].

$$\begin{array}{c} \mathrm{UA}+H_{2}O+O_{2}\overset{\mathrm{uricase}}{⟶}\mathrm{Allantoin}+CO_{2}+H_{2}O_{2}\\ H_{2}O_{2}⟶O_{2}+2H^{+}+2e^{-} \end{array}$$

The cyclic-voltammetry test of the fabricated biosensor were performed in PBS solution (pH 7.2) at a scanning rate of 0.1 V/s in the potential range of −0.2∼0.8 V. The CV curves can reflect the oxidization and reduction reaction that occur on the surface of the electrodes.

**Figure 4:**
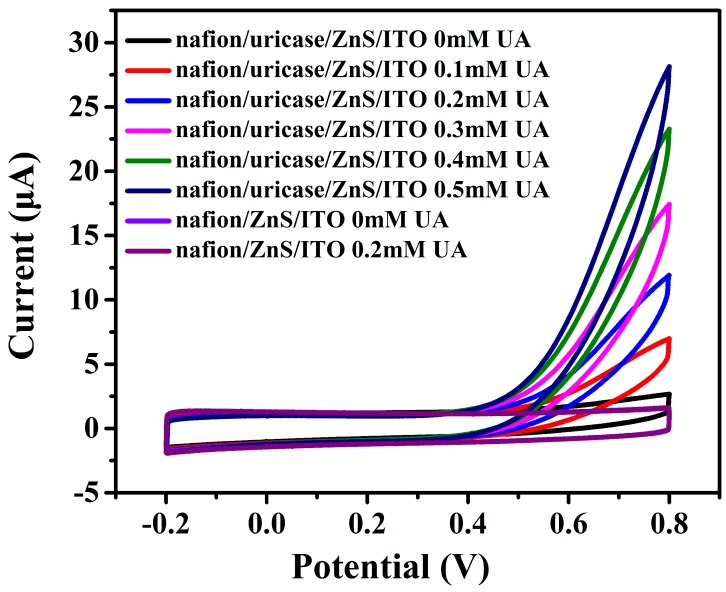
Cyclic-voltammetry results using nafion/uricase/ZnS urchin-like nanostructures electrode and bare nafion/urchin-like ZnS nanocrystals/ITO electrodes.

It can be seen in Figure 4 that there were no current rising on the bare nafion/urchin-like ZnS nanocrystals/ITO electrodes with UA. However, a strong rising of current in the biosensor electrode made of nafion/uricase/ZnS urchin-like nanostructures were observed as the UA concentration increased as a consequence of the electrocatalytic oxidation of UA. The same electric response was found in ZnS nanoparticles and the nanoflakes-based electrode. This demonstrates the oxidization of uricase by the catalysis of the enzyme. ZnS nanocrystals increased the surface area for enzyme immobilization and preserve the bioactivity of the enzyme after the immobilization process. The high conductivity of the nanomaterials also facilitates the fast transfer of electrons generated from the oxidation reaction of H${}_{2}$O${}_{2}$ between the anchored enzyme and the conducting substrate.

**Figure 5:**
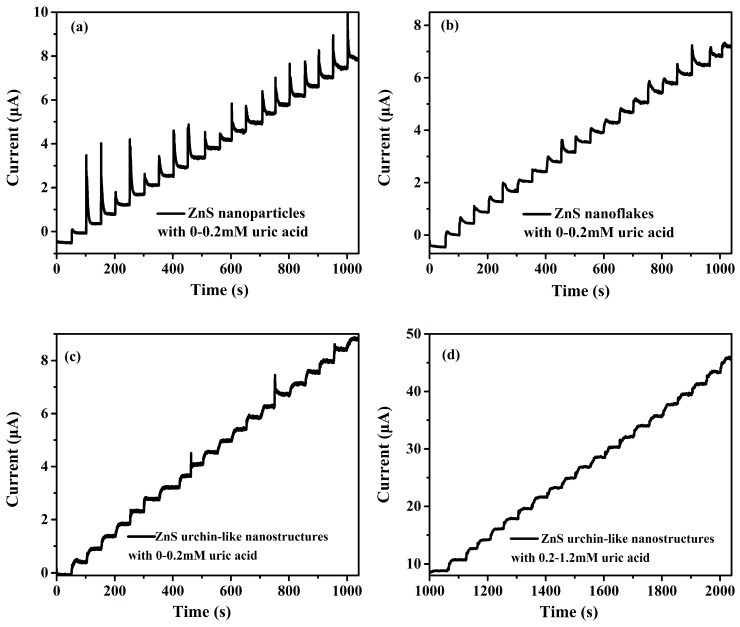
Current response curves of the nafion/uricase/nafion/ZnS nanoparticles (**a**) nanoflakes (**b**) urchin-like nanostructures (**c**,**d**)/ITO electrode after continuously adding UA.

The amperometric response for the UA of different biosensors were also measured. In Figure 5, all three biosensors showed rapid current rising at an applied potential of 0.8 V with the addition of UA in a long range under the stirring condition. Compared with nanoparticles and nanoflakes, the current stairs of ZnS urchin-like nanosensor in Figure 5c is more steady without much sharp rising and slow decreasing as shown in Figure 5a,b. The shortest current balance time is 9 s on average in Figure 5c, 13 s in Figure 5a and 17 s in Figure 5b. At a higher concentration beyond the detection linear range, the current became unstable and saturated.

**Figure 6:**
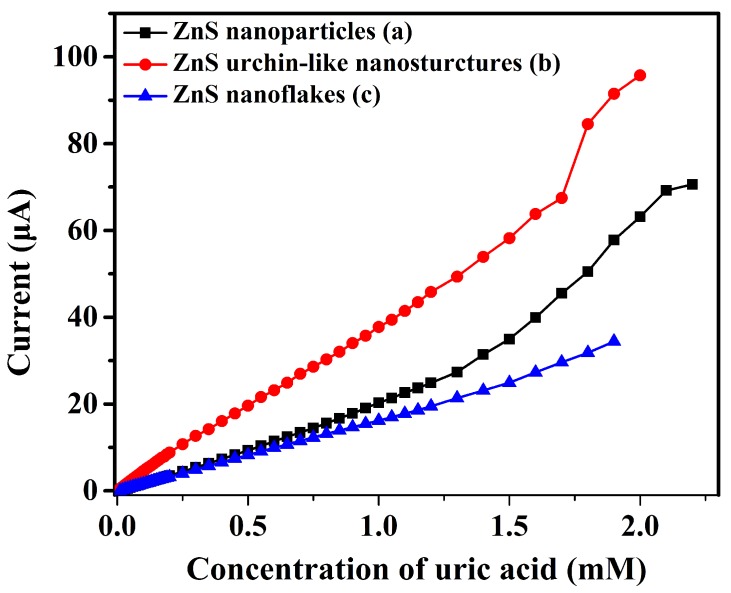
The relation between measured current and the concentration of uric acid (UA).

The current-to-concentration calibration curves of the UA biosensors fabricated with different ZnS nanostructures were shown in Figure 6. The sensing properties such as sensitivity and linear range can be judged visually from the calibration curves. Detailed performance of the sensors were calculated and shown in Table 1. The linear equations are respectively (a) $y=21.59\times 10^{-6}x-8.2009\times 10^{-7}$, $R^{2}=0.99323$; (b) $y=38.06\times 10^{-6}x-5.8171\times 10^{-7}$, $R^{2}=0.99855$; (c) $y=17.14\times 10^{-6}x-3.09466\times \;10^{-7},R^{2}=0.99707$, where *y* and *x* stand for current (A) and the concentration (mM) of UA separately. The biosensor made using ZnS urchin-like nanostructures shows a best sensitivity of 76.12 μA· cm${}^{-2}$· mM${}^{-1}$ and a lowest detection limit due to its three-dimensional morphology and hence a larger surface-to-volume-ratio that enables a better immobilization of the enzyme.

### 3.3. Photoelectrochemical Measurements

ZnS nanostructures possessed photoelectrochemical property for its potential application in photoelectric biosensors [21,30]. Electron-hole pairs were yielded in the conduction band and the valence-band with irradiation of light upon ZnS nanostructures. The transfer of electrons and holes between nanomaterials and substrate could enhance redox processes on the electrode [31]. Therefore, the photoelectrochemical UA detection by enzymatic ZnS nanosensor was also studied and discussed.

**Figure 7:**
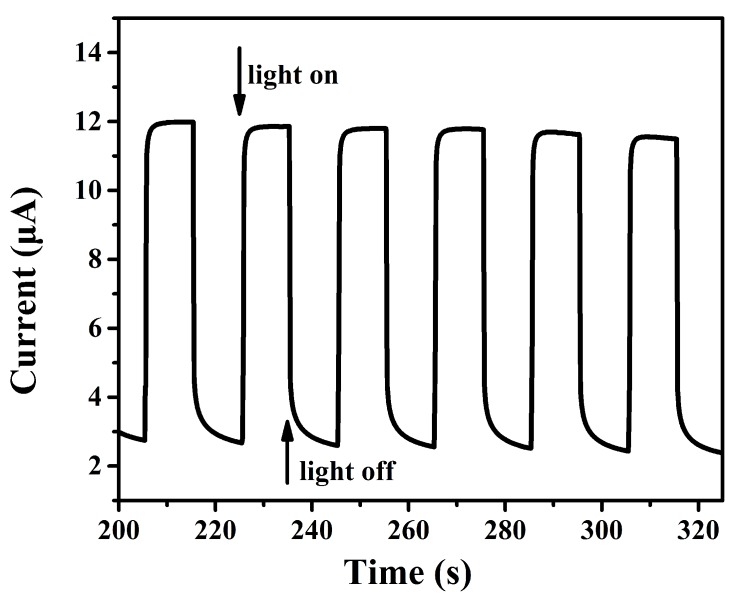
Photocurrent response of the nafion/uricase/ZnS urchin-like nanostructures/ITO electrode in phosphate buffer solution (pH 7.2) with an applied potential of 0.8V.

Figure 7 shows the photocurrent response of Nafion/uricase/ZnS urchin-like nanostructures/ITO electrode with and without the illumination of UV light with wavelength of 300 nm. The photocurrent increased quickly to 90% of the highest value within 0.4 s upon irradiation and then returned back to its previous position in a short time of 1.2 s when the UV light was off. The ratio of $I_{light}/I_{dark}$ was 4.3 and the photocurrent response was stable and repeatable. No current rising was observed on electrode without ZnS nanostructrues upon irradiation, which proved that the photocurrent were derived by ZnS nanostructures.

**Figure 8:**
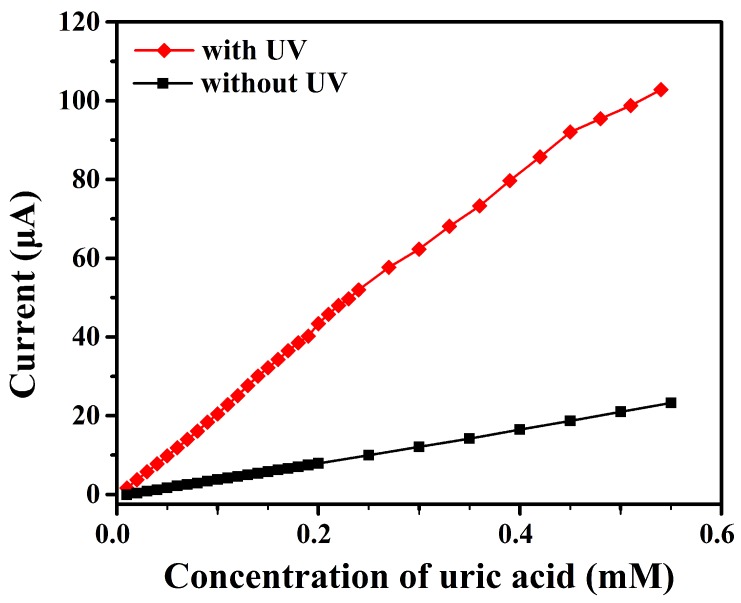
The relation between measured current and the UA concentration using a nafion/uricase/ZnS urchin-like nanostructures/ITO electrode with 300 nm UV light on and light off.

The photoelectrochemical UA detection was the same as electrochemical method with one exception of the 300 nm UV irradiation. The working electrode was tested while exposed to the illumination of a 200 W mercury lamp. The photocurrent also increased linearly with the addition of UA. The photocurrent response were about 5 times higher than that without irradiation when same amount of UA were added (Figure 8). The sensitivity of UA determination under UV light exposure was 413.98 μA·cm${}^{-2}$·mM${}^{-1}$, while the linear range was 0.01 μM–0.55 μM, lower than that without illumination. The detection limit was 0.045 μM. The theory proposed by Sun can be referred here to explain the mechanism of photoelectrochemical sensing of UA by nafion/uricase/ZnS/ITO electrode as follows [31]: Electron and hole pairs were generated with irradiation due to the photoelectrochemical property of ZnS nanostructures. The intermediate product H${}_{2}$O${}_{2}$ was oxidized by the valence-band hole and generated an electrochemical current while the conduction-band electron was transferred to the electrode to produce a photocurrent. The current response with irradiation was actually composed of electrochemical current and photocurrent. So, the ZnS biosensor was more responsive towards UA than without illumination.

### 3.4. Stability and Selectivity Test

**Figure 9:**
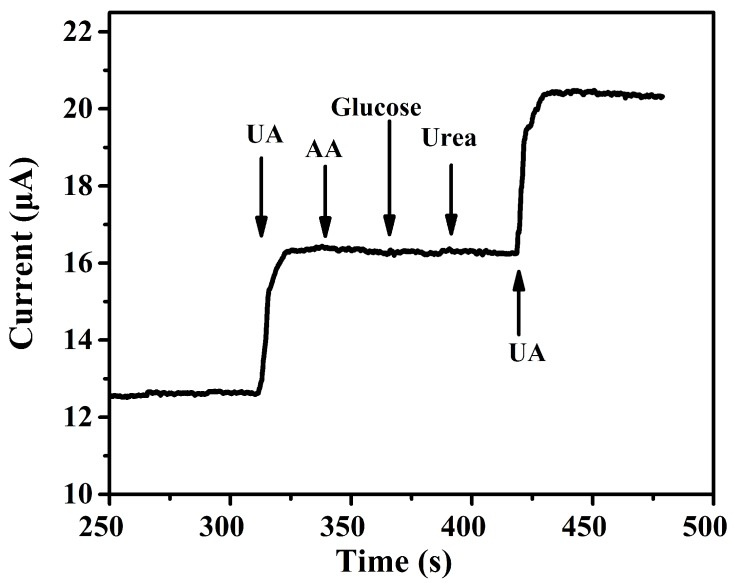
Interference tests of the biosensor upon addition of UA (UA, 0.2 mM) AA (0.2 mM), glucose (0.2 mM) and Urea (0.2 mM) in 50 mM PBS (pH 7.2).

The storage stability of this UA biosensor was investigated. The sensors were stored in the fridge at 4 ${}^{\circ }$C for four weeks and taken out for measurements of UA at the same condition per week. The biosensor retained at least 90% of the original current response after thirty days storage, which proved the high stability over time for UA determination. The results indicated the stable property of the immobilization and activity of enzyme on the electrode. The selectivity of the biosensor was evaluated by measuring amperometric response with 0.2 mM UA and 0.2 mM common interference as AA, Glucose and Urea. The result in Figure 9 shows that the appearance of these interferences apparently do not affect the current response. So the fabricated ZnS nanostructures electrodes can be used as stable and efficient biosensors for electrochemical and photoelectrochemical UA detection.

## 4. Conclusions

ZnS nanomaterials of different morphologies were synthesized through a simple hydrothermal method. With a spin-coating method, these nanomaterials were fabricated into Nafion/uricase/ZnS/ITO electrodes which have been successfully applied for electrochemical and photoelectrochemical UA detection in aqueous solution. The ZnS urchin-like nanostructures based electrochemical biosensor showed the highest sensitivity of 76.12 μA·cm${}^{-2}$· mM${}^{-1}$. The wide linear detection range from 0.01 mM to 1.7 mM also meets the requirements for diagnosis in clinic. With irradiation of 300 nm UV, its sensitivity of detecting UA is increased 5 times, reaching 413.98 μA·cm${}^{-2}$· mM${}^{-1}$, which is higher than that of most electrochemical biosensors. However, the linear range is decreased to 0.01–0.54 mM, which is to be improved in future work. These results provide a simple and efficient approach to fabricate ZnS based electrochemical and photoelectrochemical biosensors.

## Figures and Tables

**Table 1:** Camparation of recently reported UA biosensors and the units for sensitivity, limitation of determination (LOD) and linear range are respectively μA·cm${}^{-2}$·mM${}^{-1}$, μM and mM.

Materials	Method	Sensitivity	LOD	Linear Range	Reference
ZnS nanoparticles	electrochemical	43.18	1.79	0.01−1.5	This work
ZnS nanoflakes	electrochemical	34.28	1.51	0.01−2.0	This work
ZnS urchin-like nanostructures	electrochemical	76.12	0.7	0.01−1.7	This work
ZnS urchin-like nanostructures	photoelectrochemcial	413.98	0.045	0.01−0.54	This work
ZnO nanosheets	electrochemical	129.81	0.019	0.05−2.0	[5]
ZnS quantum dots	photoluminescence	-	2	0.005−2	[6]
ZnO thin films	electrochemical	1100	-	0.05−1.0	[7]
BS3/APTES	electrochemical	46.26	8.4	0.058−0.71	[8]
ZnO micro/nano wires	electrochemical	89.74	25.6	0.1−0.59	[9]
RGO-ZnO/GCE	electrochemical	109.75	0.312	0.001−0.8	[10]
RGO-ZnO/GCE	electrochemical	161.2	0.46	0.001−0.08	[11]
HCNTs/GCE	electrochemical	-	1.5	0.0067−0.065	[12]
